# Sodium butyrate enhances titanium nail osseointegration in ovariectomized rats by inhibiting the PKCα/NOX4/ROS/NF-κB pathways

**DOI:** 10.1186/s13018-023-04013-y

**Published:** 2023-08-01

**Authors:** Zhiyi Liu, Xuewei Yao, Wenkai Jiang, Zhi Zhou, Min Yang

**Affiliations:** Department of Trauma Orthopedics, The First Affiliated Hospital of Wannan Medical College, Yijishan Hospital, Wuhu, Anhui 241001 People’s Republic of China

**Keywords:** Sodium butyrate, Osteoporosis, Postoperative osteoporotic fractures, Titanium nail osseointegration, ROS, PKCα/NOX4/ROS/NF-κB pathways

## Abstract

**Background:**

Elevated levels of oxidative stress as a consequence of estrogen deficiency serve as a key driver of the onset of osteoporosis (OP). In addition to increasing the risk of bone fractures, OP can reduce the bone volume proximal to titanium nails implanted to treat these osteoporotic fractures, thereby contributing to titanium nail loosening. Sodium butyrate (NaB) is a short-chain fatty acid produced by members of the gut microbiota that exhibits robust antioxidant and anti-inflammatory properties.

**Methods:**

OP fracture model rats parameters including bone mineral density (BMD), new bone formation, and the number of bonelets around the implanted nail were analyzed via micro-CT scans, H&E staining, and Masson’s staining. The protective effects of NaB on such osseointegration and the underlying mechanisms were further studied in vitro using MC3T3-E1 cells treated with carbonyl cyanide m-chlorophenylhydrazone (CCCP) to induce oxidative stress. Techniques including Western immunoblotting, electron microscopy, flow cytometry, alkaline phosphatase (ALP) staining, and osteoblast mineralization assays were employed to probe behaviors such as reactive oxygen species production, mineralization activity, ALP activity, protein expression, and the ability of cells to attach to and survive on titanium plates.

**Results:**

NaB treatment was found to enhance ALP activity, mineralization capacity, and Coll-I, BMP2, and OCN expression levels in CCCP-treated MC3T3-E1 cells, while also suppressing PKC and NF-κB expression and enhancing Nrf2 and HO-1 expression in these cells. NaB further suppressed intracellular ROS production and malondialdehyde levels within the cytosol while enhancing superoxide dismutase activity and lowering the apoptotic death rate. In line with these results, in vivo work revealed an increase in BMD in NaB-treated rats that was associated with enhanced bone formation surrounding titanium nails.

**Conclusion:**

These findings indicate that NaB may represent a valuable compound that can be postoperatively administered to aid in treating OP fractures through the enhancement of titanium nail osseointegration.

## Introduction

Osteoporosis (OP) is a form of progressive, chronic, degenerative bone disease that is highly prevalent among older adults [1]. OP causes a dramatic loss of bone mineral density (BMD) and bone mass together with the destruction of bone microstructural integrity, contributing to greater bone fragility and a consequently elevated risk of nonviolent or low-energy fractures [2, 2]. OP can have a severe adverse impact on the quality of life of affected patients, who are primarily middle-aged and older women, thus imposing a substantial burden on the healthcare system and society as a whole [4]. Following the surgical repair of bone fractures in postmenopausal OP (PMO) patients, micro-loosening between the bone and the implanted titanium nail is a common cause of surgical failure [5–8]. Conversely, the presence of sufficient bone surrounding the implanted nail is an important predictor of successful surgical outcomes [9]^9^. Traditional pharmacological interventions deployed to improve the bone mass of PMO patients have included bisphosphonates [11, 11] but recent evidence suggests that prolonged bisphosphonate use can result in adverse outcomes including impaired angiogenesis and jaw osteonecrosis, thereby ultimately worsening implant osseointegration [13–15]. There is thus a clear need for the design of new therapeutic strategies that can enhance titanium nail osseointegration in PMO patients following fracture surgery.

The mechanisms that drive the progression of PMO and associated fracture risk are complex and multifactorial. However, oxidative stress (OS) has been repeatedly established as an important pathogenic factor associated with OP owing to its ability to suppress the proliferation and differentiation of osteoblasts [16] while also driving enhanced osteoclast proliferation [17–20]. Overly high levels of reactive oxygen species (ROS) can contribute to osteoblastic mitochondrial dysfunction, thereby impairing the physiological activity of these cells, decreasing the expression of osteogenic factors such as BMP2 and thereby compromising the process of osteogenesis [21, 21]. Normally, ROS production is counteracted by cellular antioxidant systems that keep OS in check. However, when this dynamic ROS homeostasis becomes dysregulated, levels of antioxidant enzymes such as superoxide dismutase (SOD) may decrease while malondialdehyde (MDA) levels can increase, reflecting an overall rise in OS levels that have a deleterious impact on osteoblasts [23, 23]. In time, this progressive inhibitory activity contributes to sparser bone trabeculae, decreased bone density, lower amounts of bone surrounding implanted titanium nails, and a higher risk of titanium nail loosening [25].

The gut-bone axis has emerged as an increasingly well-documented pathway, with many studies having suggested that probiotic factors and metabolites derived from the intestines can ultimately contribute to improvements in bone mass [26–28]. Sodium butyrate (NaB) is a short-chain fatty acid sodium salt generated by gut bacteria through the process of anaerobic fermentation. NaB can rapidly supply energy to local cells within the intestine while also driving enhanced probiotic microbial growth [29], and exerting antioxidant and anti-inflammatory effects within the intestines and systemically [30]. NaB serves as a histone deacetylase (HDAC) inhibitor and has been reported to induce cancer cell apoptotic death [31]. However, NaB can also reportedly facilitate more robust osteoblast proliferation and metabolic functionality, contributing to improved alkaline phosphatase (ALP) activity [32], bone mineralizing nodule production [33], and decreased bone loss through mechanisms dependent upon the Nrf2 signaling pathway [34]. NaB can also decrease protein kinase C (PKC) expression in noncancerous cells [35], counteracting the PKC-NADPH oxidase activation, which decrease ROS production, lipid peroxidation, and associated cellular damage [36, 36]. Elevated PKC protein levels can suppress intra-membrane osteogenesis and spur increased ROS generation, particularly in the presence of fosetyl myristate acetate (PMA/TPA) [38]. The NOX4 oxidase is expressed in the bone tissue and serves as a direct mediator of ROS generation downstream of PKC, thereby exacerbating the apoptotic death of osteoblasts [39]. Classical NF-κB signaling pathway activation has also been linked to inflammatory activity and OS, simultaneously impairing osteoblast survival and enhancing osteoclast function [40]. PKC can activate NF-κB signaling activity [41, 41]. NaB exerts anti-inflammatory activity in a range of settings [43]. As such, NaB may help to protect against OS-related damage in osteoblasts through the PKC and NF-κB pathways, facilitating improved improve titanium nail osseointegration following PMO fracture surgery.

This study was designed to explore the ability of NaB to reduce bone loss surrounding titanium nails following PMO fracture surgery, and to use MC3T3-E1 cells to establish an in vitro model of OS in osteoblasts in an effort to more fully understand how NaB can facilitate enhanced osseointegration in the context of PMO fracture surgery.

## Materials and methods

### Animal model groups

Female SD rats (*n* = 60, 220 ± 20 g, 8 weeks old) were obtained from Qinglong Shan (Nanjing, China). Rats were randomized into a normal group (*n* = 20) and a model group (*n* = 40). After a 1-week acclimatization period at Yiji Shan Hospital in standard cages under controlled conditions (22 ± 2 °C, 50% humidity). Rats had free food and water access at all times. Model group rats were ovariectomized (OVX), while rats in the sham control group had an equal amount of fat surrounding the ovaries removed. For 3 days post-surgery, rats were intramuscularly treated with ceftiofur sodium. During the feeding period, the rats lived in standard feeding cages without any jumping exercises [44]. After 3 months with weekly recordings of animal body weight, the femoral trunks were isolated from 5 randomly selected rats in the control and model groups for micro-CT analyses of bone trabeculae and H&E staining to confirm the successful establishment of a POM model. Of the remaining rats, animals in the sham surgery group (*n* = 15) were implanted with a titanium nail (1.5 mm × 10 mm; TA1; Huachuang Medical Devices Co., Ltd.) in the distal femur, while the model group was separated into an OVX-Ti group (*n* = 15), and an OVX-Ti + NaB group (NaB; Beyotime, China) group (*n* = 15) following nail implantation, and all of the nail implantation groups were routinely infected for 3 days postoperatively. Rats in the OVX-Ti + NaB group were gavaged with 280 mg/kg NaB, while OVX-Ti group rats instead received an equal volume of saline. Rats were treated daily for 30 days.

### Evaluation of bone volume and microarchitecture

To assess the bone volume surrounding titanium nails, the rat femoral stem was excised and fixed using 4% paraformaldehyde (Beyotime, China), and the distal end was then scanned with a Micro-CT scanner (Scanco, Switzerland) to detect bone trabeculae, with analyzed parameters including BMD, bone density, and relative bone trabecular volume fraction (BV/TV) being assessed around the titanium nail.

### Titanium nail pullout force assay

Titanium nail stability in bone was evaluated by removing fibrous tissue surrounding the nail and then utilizing a loading device to secure this nail and the femoral stem. The level of axial force necessary to pull the titanium nail out was then measured with a torque of 15 N/mm being used to pull the nail outward until it had been fully removed, with the final force being recorded.

### Staining of pathological tissue sections

An EDTA decalcification solution (pH 7.2–7.4) was used to decalcify femoral stem samples for 1 month, with the solution being replaced every third day. Following complete decalcification, femoral stem samples were dehydrated, embedded in paraffin, cut into sections, blotted, and sealed for processing. Bony trabeculae numbers surrounding the titanium nail were determined through H&E staining, while a Masson’s staining kit (Solarbio, USA) was used to stain collagen and muscle fibers (blue and red, respectively), with collagen fibers being a characteristic indicator of new and mature bone. An osteoclast staining kit (Servicebio, China) was used to stain bone tissue sections to reveal clear vacuoles surrounding the bone trabeculae and red-stained osteoclasts attached to the bone.

### Cell culture and treatment conditions

The MC3T3-E1 subclonal 14 preosteoblast cell line was obtained from the Cellcook Corporation cell ban. Cells were cultured in α-MEM (Gibco; USA) at 37 °C in a 5% CO2 incubator, with media being exchanged every other day and cells being passaged with 80% confluent. Oxidative stress was induced in these cells by treating them with carbonylcyanide-m-chlorophenylhydrazone (CCCP; Solarbio, USA), while phorbol-12-myristate-13-acetate (PMA/TPA; Beyotime, China) was used to activate the PKC protein pathway and NaB was used to inhibit.

MC3T3-E1 cell basal media consisted of α-MEM containing 10% fetal bovine serum (FBS; Gibco, USA) and 1% penicillin/streptomycin (Beyotime, China). Osteogenic mineralization induction medium instead contained 10% FBS, 1% penicillin–streptomycin, 10 mM β-glycerophosphate (Sigma, USA), 50 μM ascorbic acid (Solarbio, USA), and 100 nM dexamethasone (Solarbio, USA).

For cells cultured in the absence of titanium plates, cells were assigned to the normal, CCCP, CCCP+NaB, and CCCP+NaB+TPA treatment groups. Briefly, these MC3T3-E1 cells were added to 24-well (3 × 10^4^/well) or 6-well (10 × 10^4^/well) plates, with media being exchanged every 2 days. For culture on titanium plates, cells were assigned to normal, CCCP, CCCP+NaB, and CCCP+NaB+TPA groups, and were plated in 6-well plates (10 × 10^4^/well). Media was exchanged every 2 days, and the quantification and microscopic examination of cells in the discarded supernatant fraction was performed. Cell treatments in the established groups are defined below.

Normal group: osteogenic culture medium for 7 days; CCCP group: osteogenic culture medium for 6 days following stimulation for 1 h with CCCP (10 μM) and replacement with 10 μM CCCP medium for 24 h; CCCP+NaB group: osteogenic culture medium for 6 days following stimulation with 10 μM CCCP for 1 h and replacement with 10 μM CCCP + 0.3 mM NaB medium for 24 h; CCCP+NaB+TPA group: cells were stimulated with 10 μM CCCP for 1 h following 6 days of culture in osteogenic medium, media was exchanged for media containing 0.1 nM TPA+10 μM CCCP for 4 and was then replaced for 24 h with media containing 10 μM CCCP+0.3 mM NaB.

### Analyses of cell viability

A Cell Viability Counting Kit (CCK8; Solarbio, USA) was used to monitor MC3T3-E1 cell proliferation in response to NaB and TPA treatment. Briefly, these cells were added to 96-well plates (3 × 10^3^/well in 100 μL) and treated with a range of NaB (0.1–0.9 mM) and TPA (0.1–0.9 mM) concentrations at 37 °C in a tissue culture incubator. After 24, 48, or 72 h, 10 ul of CCK8 reagent was added per well, and plates were incubated for an additional 2 h. Absorbance at 450 nm was then measured via a microplate reader (BioTek Instruments, Inc., USA).

### ALP staining and activity assays

The effects of NaB on the osteogenic differentiation of CCCP-treated cells were analyzed through ALP staining for cells cultured in the absence of titanium plates. After culture for 7 days(6 days in osteogenic media + 1 day in group treatment), cells were fixed for 20 min using 4% paraformaldehyde (Beyotime, China) and stained with a BCIP/NBT staining working solution (Beyotime, China), for 30 min at room temperature in the dark. Color development was then stopped, and cells were imaged via light microscopy (Nikon eclipse Ti-U; USA). ALP activity levels were measured with an ALP activity assay kit (Beyotime, China) based on provided instructions.

### Alizarin red staining and mineralization assays

The impact of NaB on the osteogenic mineralization capacity of CCCP-treated osteoblasts was evaluated via alizarin red staining in titanium-free plates on day 22 (21 days in osteogenic media + 1 day in group treatment). On day 22, cells were fixed for 20 min, and osteoblast mineralized nodules were treated with alizarin red S staining solution (Beyotime, China) for 30 min at room temperature. Nodules were then rinsed with PBS and imaged via microscopy. To quantify mineralized nodule formation, at 2 h post-staining, cells were treated for 1 h with 10% cetyl pyridine chloride (Solarbio, USA), after which absorbance at 570 nm was measured.

### Scanning electron microscopy (SEM)

MC3T3-E1 cells were cultured on titanium plates (Diameter 34 mm, TA1, Baoji Titanium Co.) for 7 days(6 days in osteogenic media + 1 day in group treatment), after which they were treated with an electron microscopy fixative (Servicebio, China), at 4 °C, dehydrated with an alcohol gradient (30%, 50%, 70%, 80%, 90%, 95%, 100%, 100%, 15 min each), treated for 15 min with isoamyl acetate, and dried using a critical point drier (Quorum, UK). After spraying for 30 s with gold using a centrifugal sputterer (Hitachi, Japan), cells were imaged with a scanning electron microscope (Hitachi, Japan).

### Analyses of ROS production and redox status

ROS levels were analyzed in MC3T3-E1 cells incubated on titanium plates, with cells having been cultured and stimulated as in ALP activity assays. After 7 days, cells were rinsed with PBS, harvested, and stained for 30 min using 10 uM of 2',7'-dichlorodihydrofluorescein diacetate (DCFH-DA). After three washes, cells were suspended in serum-free media and analyzed with a flow cytometer (FC500MPL, Beckman Coulter, USA). A ROS detection kit (Beyotime, China) was used to analyze cells that were not cultured on titanium plates, with cells being imaged via a fluorescence microscope (Nikon eclipse Ti-U; USA).

### Analyses of SOD content and MDA levels

Cells were cultured and stimulated as above. After 7 days, they were rinsed using PBS and analyzed with SOD and MDA assay kits (Nanjing Jiancheng Institute of Biological Engineering; China) based on provided directions. Briefly, cells were lysed with an ultrasonic cell crusher (Ningbo Xinzhi Biological Technology Co.), and BCA kits (Beyotime, China) were used to measure protein levels. Absorbance values for individual samples were measured and used to compute SOD activity (U/mg protein) and MDA levels (nmol/mg protein) based on provided kit instructions.

### Apoptotic cell death analyses

Cells were cultured and stimulated as above. After 7 days, an Annexin V-FITC/PI dual-staining apoptosis detection kit (Bestbio, China) was used to evaluate apoptotic death in different clusters. Briefly, cells were washed with PBS, harvested, and stained as directed. Annexin V-positive and PI-negative cells were early apoptotic cells, whereas double-positive cells were late apoptotic cells. The cells in both of these gates were summed together to calculate overall rates of apoptosis.

### Western immunoblotting

Following induction for 7 days on titanium plants, MC3T3-E1 cells were washed, and RIPA buffer (Beyotime, China) containing phenylmethylsulfonyl fluoride (PMSF) was used to extract proteins, with a BCA assay kit being employed to measure protein levels in the resultant samples. A Total Bone Tissue Protein Extraction Kit (Invent Biotechnologies, Inc, USA) was instead used to extract proteins from samples of bone tissue. Following SDS-PAGE separation, these proteins were transferred onto PVDF membranes that were blocked for 2 h using BSA, rinsed, and incubated overnight with antibodies specific for Coll1 (1:1000, AF7001, Affinity, USA), OCN (1:1000, DF12303, Affinity, USA), BMP2 (1:1000, AF5163, Affinity, USA), BCL2 (1:1000, AF6139, USA), BAX (1:1000, WL01637, Wanlei, China) Caspase3 (1:1000, AF6311, Affinity, USA), PKC (1:1000, SC17769, Santa, USA), actin (1:10,000, T0022, Affinity, USA), Nrf2 (1:10,000, AF0639, Affinity, USA), HO1 (1:1000, AF5393, Affinity, USA), PKCα (1:1000, AF6196, Affinity, China), NFκB (1:1000, SC8008, Santa, USA), NOX4 (1:1000, SC518092, Santa, USA) at 4 °C with constant shaking. After rinsing with TBST, blots were incubated for 2 h with secondary anti-rat (1:5000, S0002, Affinity, USA) or anti-rabbit IgG (1:5000, S0001, Affinity, USA), followed by washing and the use of an ultrasensitive chemistry kit (Milibo, USA) to detect protein bands, with ImageJ being used for subsequent analysis.

## Results

### The impact of NaB and TPA on the proliferation of MC3T3-E1 cells

**Fig. 1:**
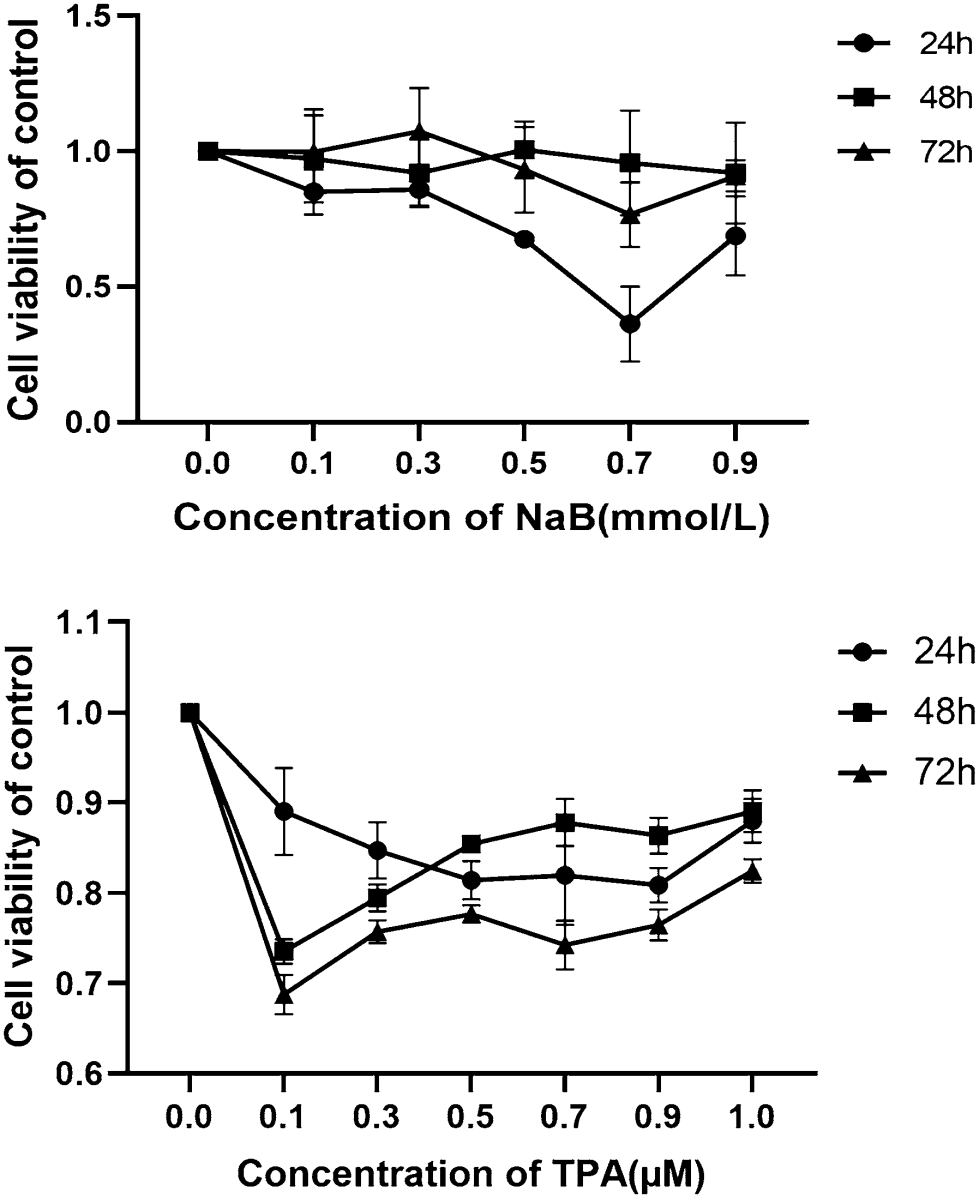
Effect of NaB and TPA on the viability of MC3T3-E1 cells. Cells MC3T3-E1 cells were treated with different concentrations (0, 0.1, 0.3, 0.5, 0.7, and 0.9 mM/L) of NaB and different concentrations (0, 0.1, 0.3, 0.5, 0.7, 0.9, 1 µM) of TPA for 24 h, 48 h, and 72 h, and cell viability was detected by CCK8 after treatment. Each experiment was repeated at least three times. Each set of data was compared with the 0 concentration group

Initially, the cytotoxicity of TPA and NaB was analyzed via CCK-8 assay, with MC3T3-E1 cells being treated with 5 different doses of these compounds for 24, 48, or 72 h. With untreated cells serving as a reference to gauge viability, a NaB dose of 0.3 mM was found to have the least inhibitory effect on proliferation after 24 h and TPA was 0.1 nM (Fig. 1).

### The impact of NaB on osteoblast function

**Fig. 2:**
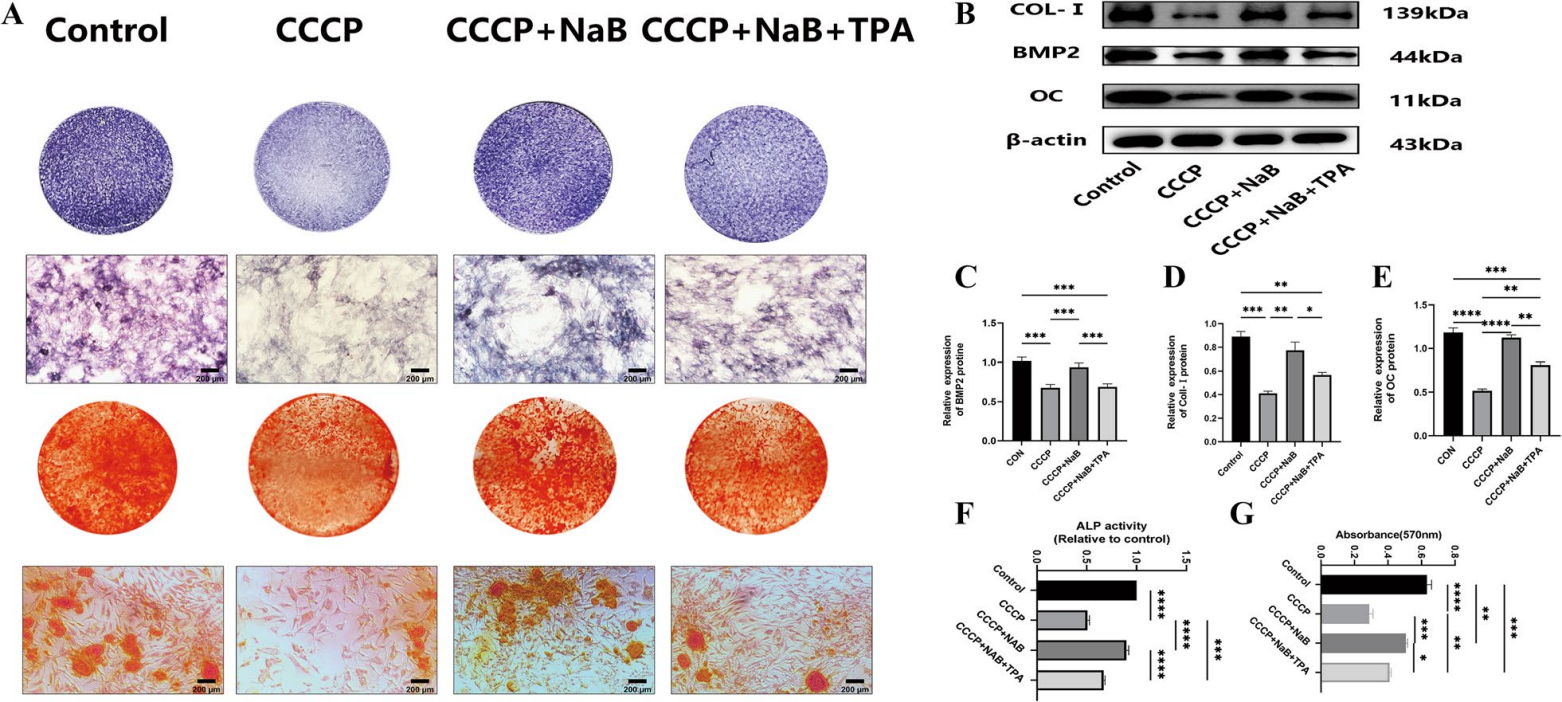
NaB promotes osteogenic differentiation and osteogenic gene expression in MC3T3-E1 cells in the CCCP-induced state. **A** Characteristic pictures of alkaline phosphatase staining and alizarin red staining, all rectangular pictures are on a scale of 200 µm. **B** western blot analysis of genes specific for osteogenic action in the titanium plate state. **C-E** Ratio of protein gray values to β-actin gray values for each group is indicated. **F** Detection of alkaline phosphatase activity in each group. **G** Assessment of absorbance of mineralized nodules in each group. Data are expressed as mean ± SEM and *P* values were calculated for each group between groups (*n* = 3) for each other, where *P* values were not significant (ns) are not shown in the graph. ^*^P < 0.05, ^**^P < 0.01,^***^p < 0.001,^****^P < 0.0001

**Fig. 3:**
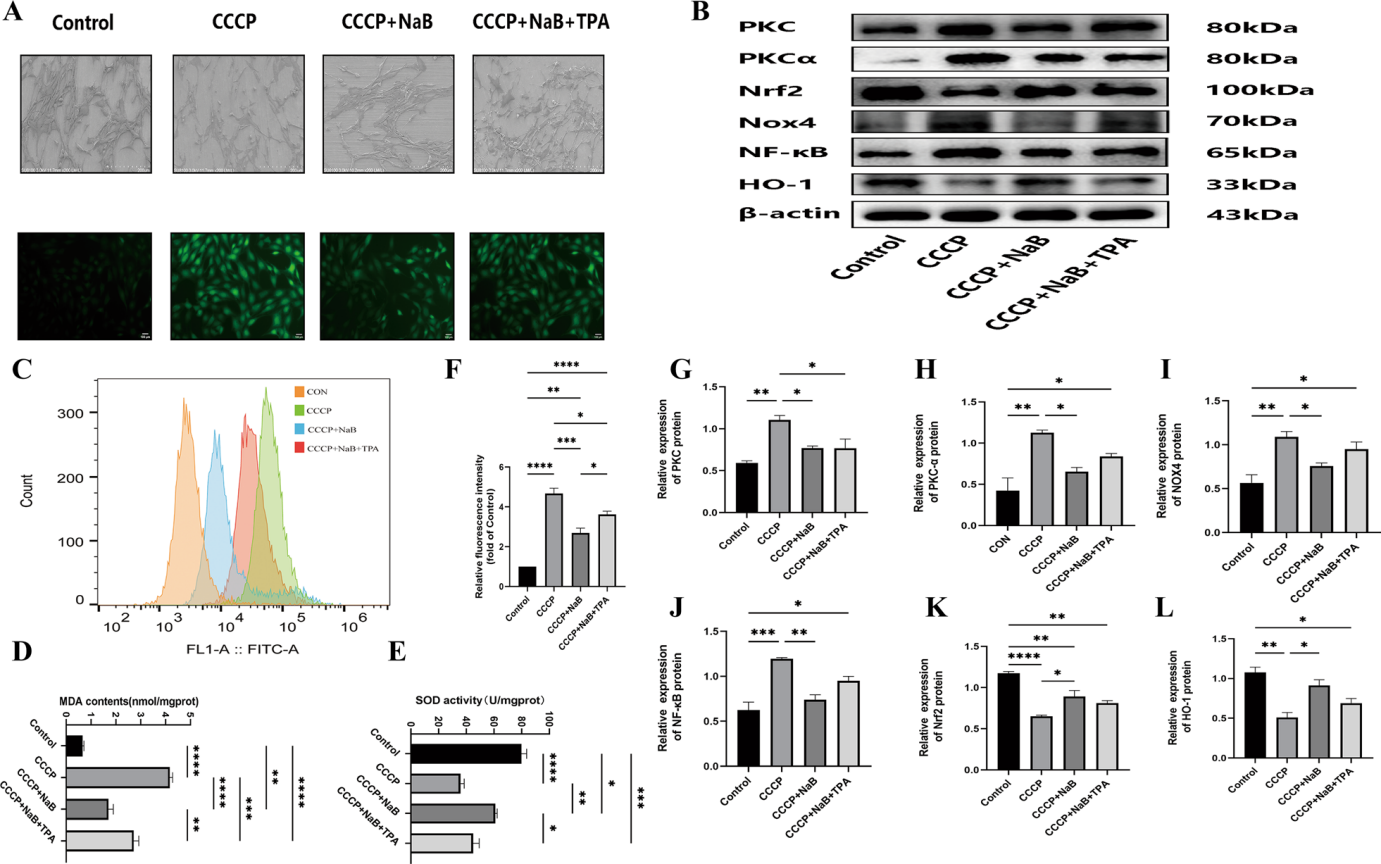
NaB enhances the antioxidant capacity of MC3T3-E1 cells by inhibiting the oxidative stress pathway. **A** Electron micrograph of MC3T3-E1 cells grown on titanium plates and fluorogram of ROS in the titanium plate free state with the scale bar of 200 µm for electron micrograph and 100 µm for fluorogram. **B** Western blot to detect the presence of target gene in the titanium plate state. **c** Flow cytometric detection of ROS in MC3T3-E1 cells in the titanium plate state. **C** western blot analysis of oxidative stress channel protein genes in the titanium plate state. **D** Expression of MDA in each group in the titanium plate state. **E** Expression of SOD in each group in the titanium plate state. **F** Ratio between fluorescence luminosity values of each group and the normal group.**G-I** Ratio between grayscale values of protein genes and β-actin, data are expressed as mean ± SEM. *P* values were calculated mutually for data between groups (*n* = 3), where *P* values were not significant (ns) are not shown in the graph. ^*^P < 0.05, ^**^P < 0.01,^***^P < 0.001,^****^P < 0.0001

To begin evaluating the effects of NaB treatment on the function of osteoblasts, MC3T3-E1 cell differentiation and mineralization were analyzed, and *osteogenesis-related gene* (*BMP2*, *OCN*, *Coll-I*) expression was assessed (Fig. 2B). This approach revealed NaB treatment to be associated with improved ALP activity, calcium deposition (Fig. 2A), and osteogenic protein expression as compared to the CCCP group although these levels remained below those in the control group. CCCP treatment was associated with increases in PKC, NOX4, and NF-κB levels together with reduced Nrf2 and HO-1 levels, while NaB normalized the PKC, NOX4, and NF-κB levels in these cells. To further test the effects of NaB on PKC signaling, the PKC agonist TPA was used in these studies, ultimately demonstrating that NaB could partially reverse the inhibitory effects of TPA treatment on ALP activity levels, the expression of osteogenic proteins, and calcium deposition. These data thus highlighted the ability of NaB to enhance osteogenic activities through PKC, NOX4, and NF-κB pathway inhibition (Fig. 3B).

### NaB induces more robust antioxidant activity in MC3T3-E1 cells

Higher OS levels in the bone tissue and impaired systemic antioxidant capacity following menopause are both important contributors to OP pathogenesis and titanium nail loosening. To examine how NaB affected the antioxidant capacity of osteoblasts, ROS (Fig. 3A), SOD(Fig. 3E), and MDA (Fig. 3D)levels in MC3T3-E1 cells were analyzed. CCCP treatment was associated with altered MC3T3-E1 cell morphology when cultured on titanium plates, together with higher intracellular ROS levels as detected via flow cytometry and fluorescence microscopy (Fig. 3C), decreased SOD activity, and higher levels of MDA, consistent with overall increases in ROS levels. NaB treatment reversed these changes, and it was also able to reverse the effects of PKC agonist treatment on ROS production with respect to both MDA levels and SOD activity. These findings suggest that NaB can modulate MC3T3-E1 cell antioxidant capacity through the inhibition of the PKC, NOX4, and NF-κB pathways (Fig. 3B).

### NaB protects MC3T3-E1 cells against CCCP-induced apoptotic death

**Fig. 4:**
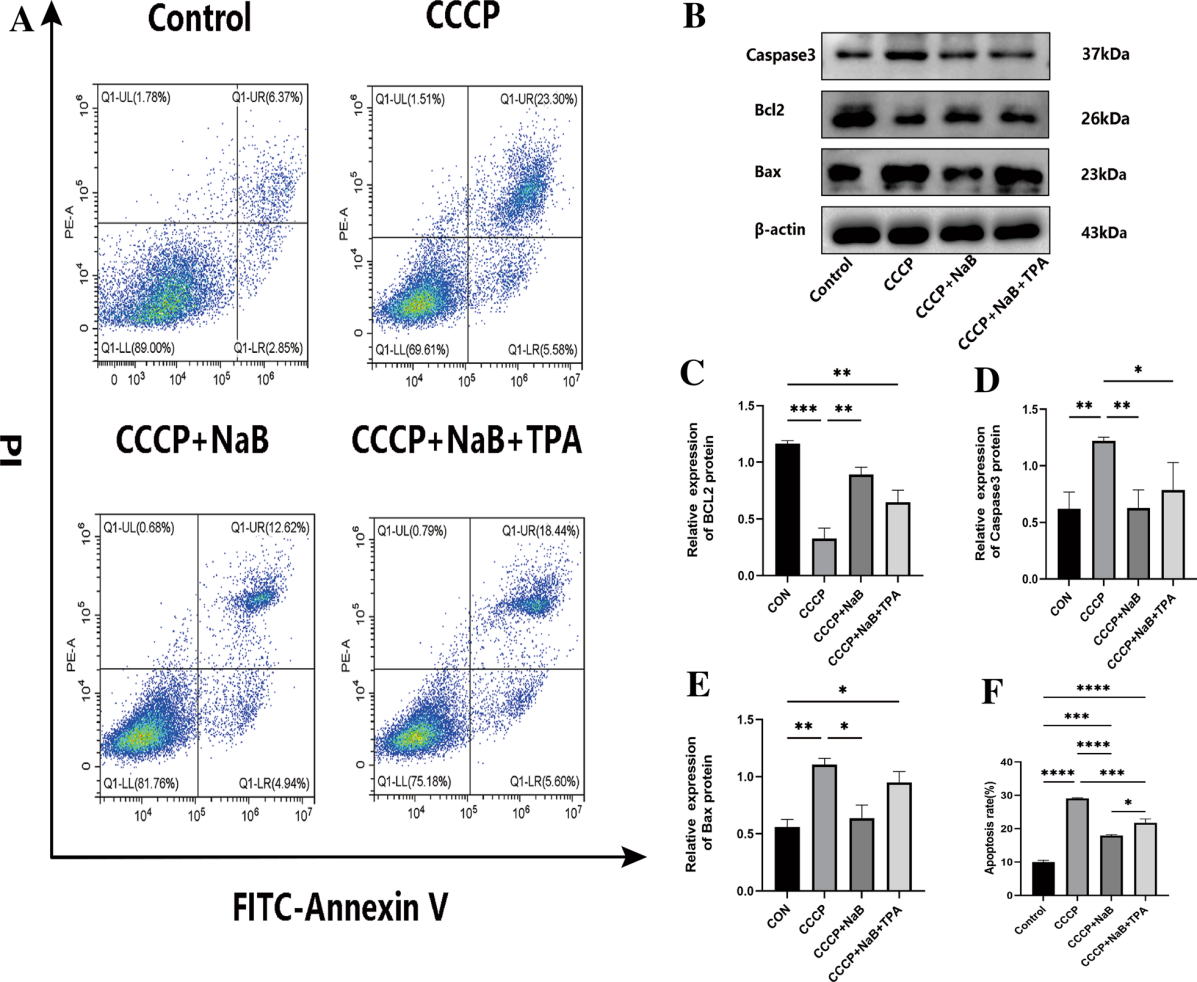
NaB reduces apoptosis in MC3T3-E1 cells stimulated by CCCP. **A** Apoptosis rate of MC3T3-E1 cells in each group in titanium plate condition. **B** Western blot detection of apoptosis-specific protein genes (Bax, Bcl2, Caspase3) expression in each group in titanium plate condition. (**C-E**) Representative grayscale values of protein display and the ratio between β- actin to each other ratio. **F** Statistical plot of apoptosis rate values for each group. Data are expressed as mean ± SEM, and *P* values were calculated mutually for data between groups (*n* = 3), where *P* values were not significant (ns) are not shown in the graph. ^*^P < 0.05, ^**^P < 0.01,^***^P < 0.001,^****^P < 0.0001

Apoptotic death can compromise osteoblast function in a postmenopausal setting, and CCCP was herein found to drive the apoptotic death of osteoblasts in vitro. Accordingly, the effects of NaB on apoptosis-related protein levels were evaluated. While CCCP was able to drive apoptosis, NaB partially rescued this effect albeit not to the levels observed in normal control cells (Fig. 4A). Specifically, NaB reduced pro-apoptotic Bax and caspase-3 levels while increasing Bcl-2 levels, with both experiments supporting the protective antiapoptotic effects of NaB (Fig. 4B).

### Rat OP model establishment

**Fig. 5:**
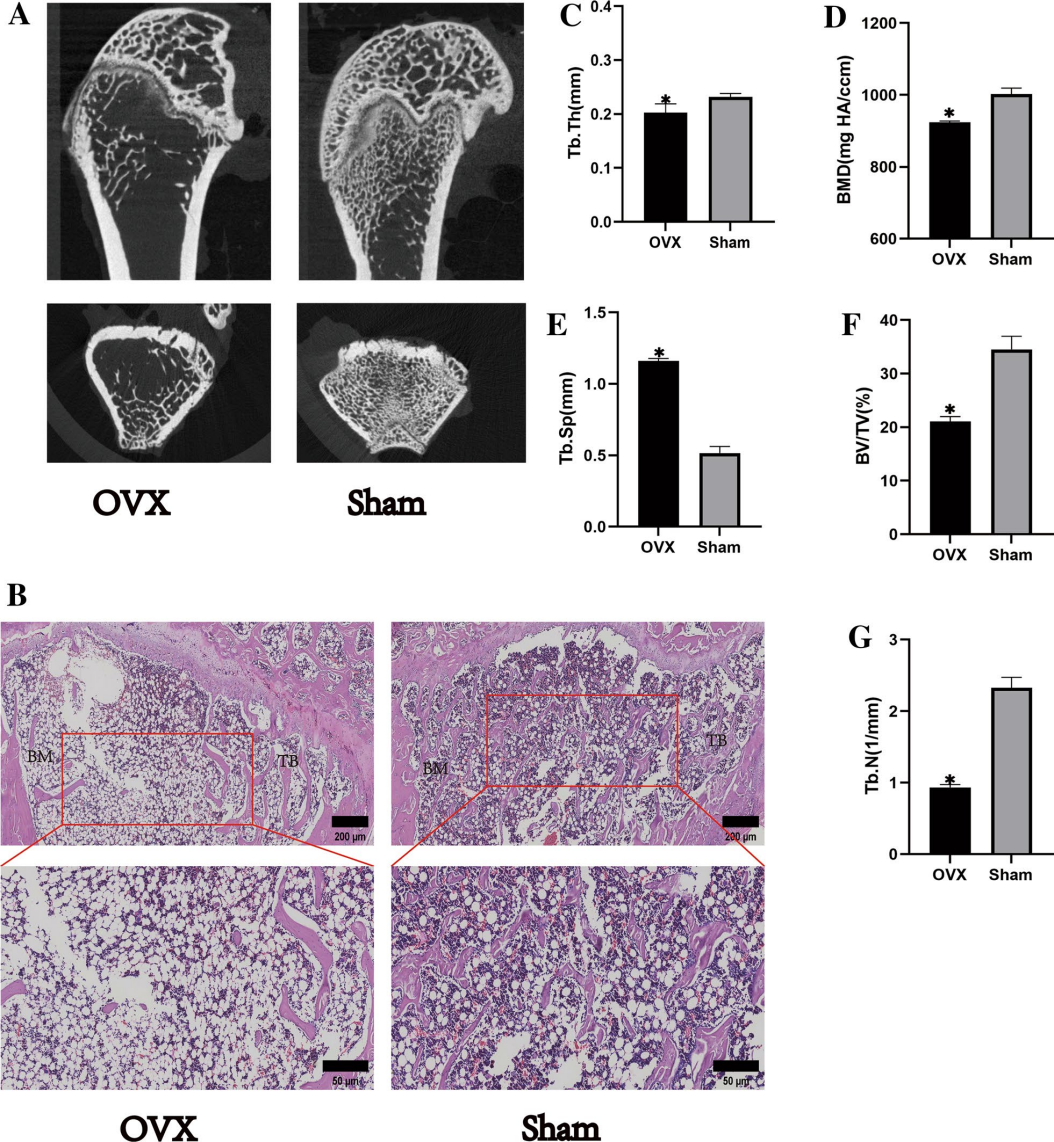
Establishment of the postmenopausal osteoporosis rat model.** A** Micro-CT scans of the distal femoral stem bone in the OVX and Sham groups. **B** HE staining of the distal femur, a,b are 5 × images and c,d are 10 × detail magnified images, both on a scale of 200 µm. (**C–G**) indicate Micro-CT assay data, respectively, bone trabecular thickness, bone mineral density, bone trabecular separation, relative bone density volume, and number of trabeculae. Data are expressed as mean ± SEM and P values were calculated between groups (*n* = 3) for each other. ^*^P < 0.05

Micro-CT scans of model rats(Fig. 5A) revealed that the trabecular thickness (Tb.Th), trabecular number (Tb.N), and bone mineral density (BMD) in the OVX group were reduced relative to the sham control group, while trabecular separation (Tb.Sp) was higher than in the sham group. H&E staining further (Fig. 5B) indicated that bone trabecular density in the OVX group was reduced relative to the sham group. These findings confirmed successful OP model establishment.

### NaB inhibits the PKC pathway to enhance bone microstructure and osseointegration in OP model rats

**Fig. 6:**
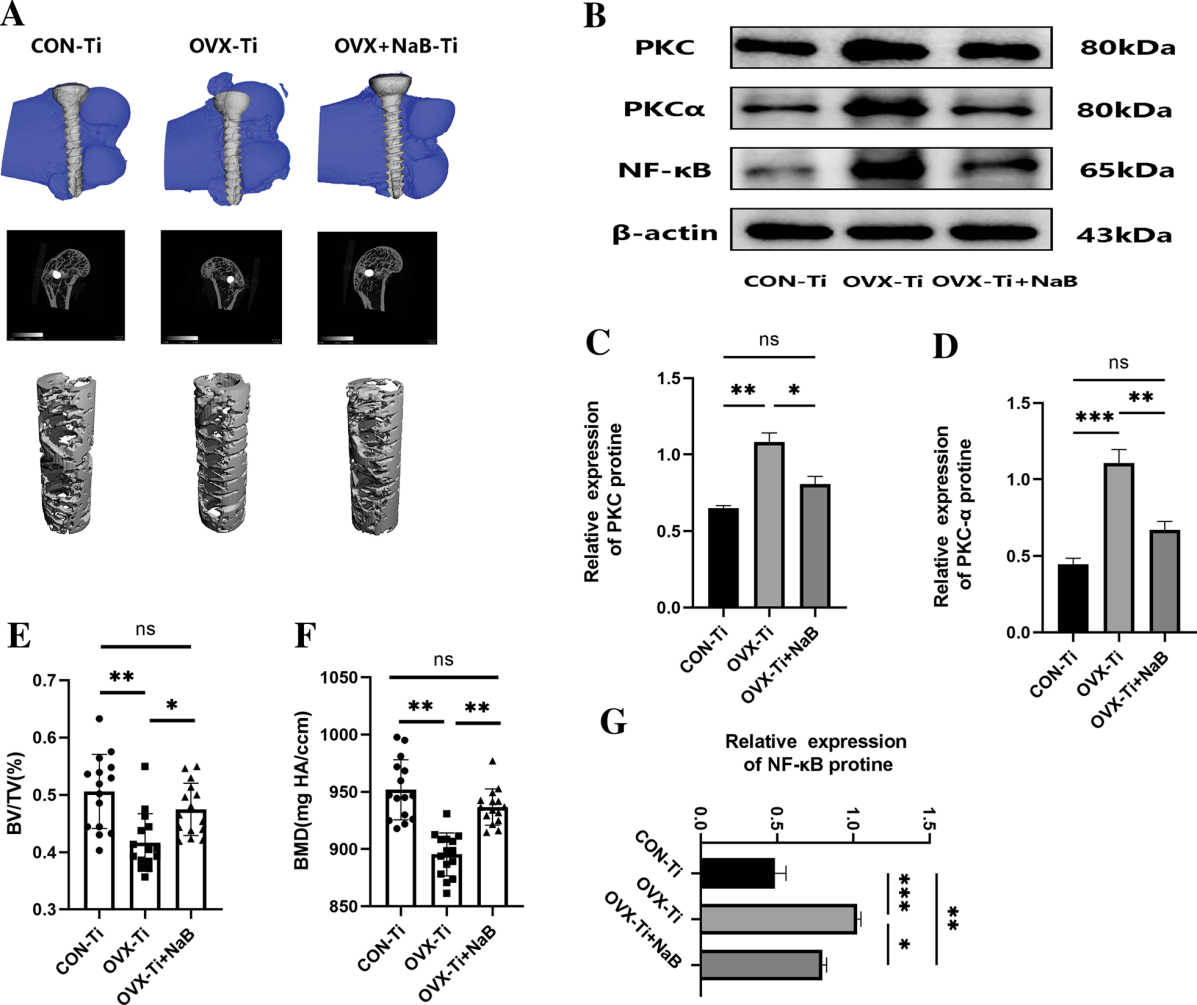
NaB enhances bone volume around titanium nail by inhibiting PKC. **A** Three-dimensional structure of titanium nail in bone, two-dimensional structure, and three-dimensional scan of bone 0.25 mm around titanium nail **B** Western blot to detect the presence of target gene in bone tissue. **E, F** Relative volume of bone trabeculae and density of bone trabeculae. **C, D, G** Ratio of gray value of target gene to β-actin. Data (*n* = 3) are expressed as mean ± SEM, and *P* values were calculated mutually between groups (*n* = 3). ^*^P < 0.05, ^**^P < 0.01,^***^P < 0.001

**Fig. 7:**
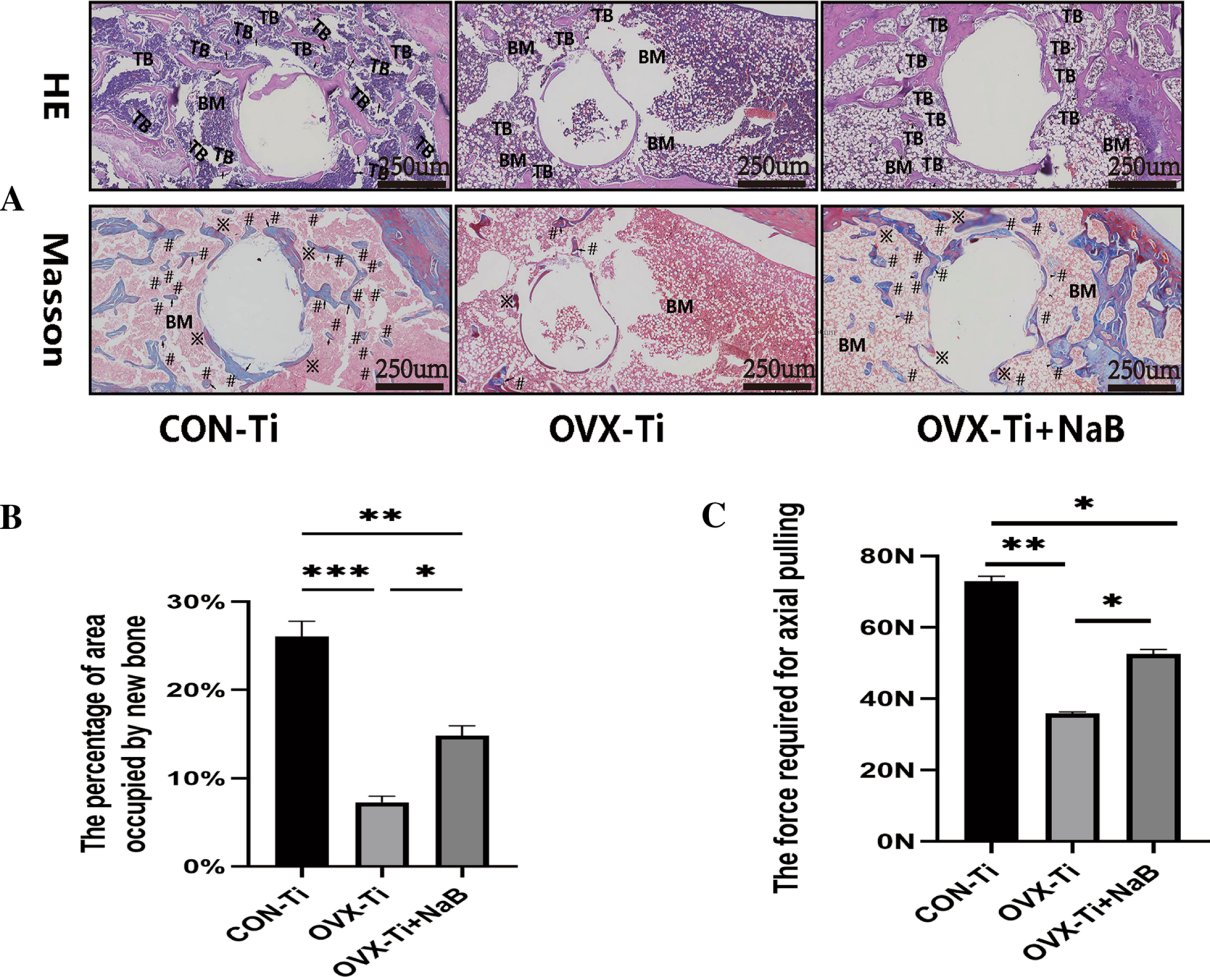
Effect of NaB on the bone around the titanium nail. **A** HE staining and Masson staining of the bone around the titanium nail, in which TB indicates bone trabeculae and BM indicates bone marrow cavity; ※represents red muscle fibers and # represents blue collagen fibers (new bone).**B** Statistical plot of the area occupied by new bone around the titanium nail. **C** Statistical plot of the axial tension required to pullout the titanium nail. Data are expressed as mean ± SEM, and *P* values were calculated for each other between groups (*n* = 3). ^*^P < 0.05, ^**^P < 0.01,^***^P < 0.001

**Fig. 8:**
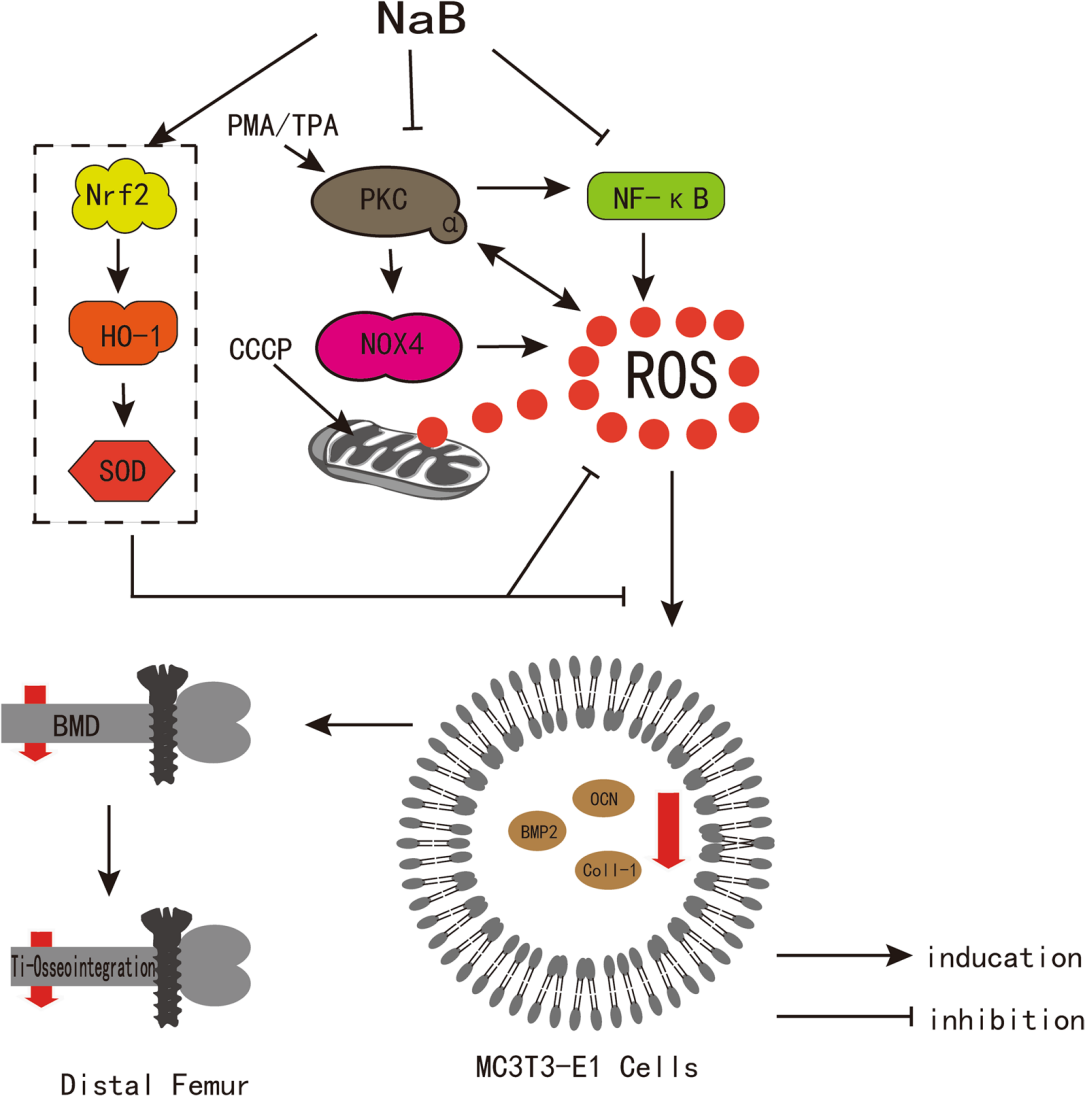
Schematic diagram summarizing the main findings in this article

Micro-CT scans of longitudinal distal femur cross sections were performed, followed by the 3D reconstruction of the 0.25 mm of bony trabeculae surrounding implanted titanium nails to examine bone microstructural characteristics in Fig. 6. In OVX model rats, bony trabeculae were sparse and clear evidence of bone microarchitectural destruction was observed surrounding the titanium nail at the distal femur end, with the bones in these rats being more brittle. Rats in the OVX-Ti+NaB treatment group, in contrast, exhibited significantly improved BMD, bone density, and bone volume fraction (BV/TV) ratio values (*P* < 0.05), consistent with the ability of NaB to restore bone density at the implant site, albeit not to control levels. Pull-out force assays similarly revealed that titanium nails were extracted most readily in the OVX-Ti group, confirming the successful simulation of the micro-loosening that impacts titanium nails after surgery in PMO fracture patients (Fig. 7C). Western immunoblotting also revealed that NaB suppressed PKC and NF-κB levels, although they remained elevated as compared to the control group, in line with the ability of NaB to restore osseointegration at least in part via PKC pathway inhibition.H&E staining indicated that higher numbers of bony trabeculae surrounding the titanium nail were evident following NaB treatment relative to the OVX-Ti group, although these levels were still below those in the normal control group. Masson’s staining for new bone indicated that following NaB treatment the titanium nail was primarily enmeshed in new bone and collagen tissue (Fig. 7A), whereas in the normal control group the nail was surrounded by collagen fibers and both new and mature bone. In the OVX-Ti group, only minimal amounts of new bone tissue were evident around the nail. The histological and micro-CT results in these experiments were therefore consistent (Fig. 8).

## Discussion

The progressive aging of the Chinese population underscores the need to focus further resources and research on geriatric diseases, with studies of the etiology of OP-related fractures having the potential to improve patient treatment options, alleviating pain and improving overall quality of life to lift this societal burden [45, 45]. A range of drugs have been used to treat OP to date, but their utility has been hampered by high prices, adverse side effects, and the fact that they cannot be used for extended periods [11] , [47]. Despite a wealth of OP-focused research in recent years, studies of the impact of NaB on osteoblasts have been limited. The loosening of implanted nails following surgical fracture treatment has frequently been observed among OP patients, and a range of biomaterial-related and biomechanical studies have demonstrated that such loosening is related to insufficient bone volume surrounding these implanted nails and a consequent lack of adhesion and osseointegration [48]. This study was designed to assess the ability of pharmacological treatment to enhance osseointegration and bone mass using in vitro and in vivo models of OP in an effort to ultimately improve patient outcomes. An OVX model was used to simulate PMO in rats, resulting in a significant loss of bone mass and impaired implant osseointegration in line with results observed in human patients. While NaB has been confirmed as a robust antioxidant capable of counteracting obesity-related OP, whether it can similarly exert benefits in the context of PMO has yet to be tested [49]. Thus, the molecular mechanisms through which NaB influences osteoblasts in the context of titanium nail osseointegration were herein explored using cultured cells and OVX model rats.

These experiments demonstrated that CCCP treatment suppressed the differentiation and mineralization capacity of MC3T3-E1 cells as evidenced by decreases in ALP activity and mineralized nodule formation. ALP secretion by osteoblasts is a major indicator of bone metabolism, with the levels of ALP being directly related to the differentiation of osteoblasts, impacting osteoblast cell matrix mineralization [50]. Mineralized nodules are a hallmark of such osteoblastic differentiation, serving as the primary morphological correlate to the function of these osteoblasts [33]. OCN levels can be analyzed to monitor the development and metabolism of bone tissue, with OCN being produced by osteoblasts during the late stages of differentiation. These OCN levels decline with age such that they serve as a valuable indicator that can be leveraged to monitor OP [51]. BMP2 regulates periosteal granule cells in the context of fracture healing and also facilitates osteoblast production within the bone marrow such that it has been employed in clinical settings [52, 52]. Collagenase type 1 (Coll-1) is a bone marker related to bony tissue growth and new bone formation, in addition to serving as a key regulator of osteoblast differentiation [54]. Here, CCCP treatment suppressed the expression of these three osteogenic proteins, whereas NaB treatment reversed these changes.

The PKC signaling pathway can, when activated, induce higher levels of OS in a range of cell types including tumor cells and vascular endothelial cells, potentially resulting in dysfunction or apoptotic death when such stress is not effectively mitigated [55, 55]. PKC is also related to the expression of the CXCR chemokine receptor family of proteins, which are important regulators of cellular proliferation [57]. NaB can regulate miR-15057 to suppress CXCR, thereby modulating CXCR protein expression and influencing antioxidant stress pathways within cells. NaB is also related to mitochondrial activity and apoptosis [58, 58]. Here, control MC3T3-E1 cells cultured on titanium plates exhibited low levels of PKC expression, whereas it was upregulated following CCCP treatment, and NaB antagonized this effect. NaB was further able to counteract the increases in PKC observed when these cells were treated using TPA, which is a PKC agonist. The NOX4 oxidase functions downstream of PKC to generate H_2_O_2_ directly without the need for additional activation steps, unlike other NOX family Excess oxidase activity can drive ROS production [60], altered mitochondrial membrane potential, reduced SOD activity, higher MDA levels, and the overall disruption of normal free radical scavenging and mitochondrial dysfunction [61]. NF-κB serves as a key transcription factor that regulates inflammatory activity, immune response induction, and apoptosis in the context of organismal stress responses [62]. Overly robust NF-κB activation can contribute to pro-apoptotic Bax upregulation and antiapoptotic Bcl-2 downregulation, contributing to the loss of bone tissue in estrogen-deficient animals. This transcription factor also regulates antioxidant proteins including NADPH dehydrogenase 1 (NQO1), and Nrf2 is an upstream target of NQO1 and HO-1 [24]. Nrf2 serves as a key regulator of antioxidant responses [63]^,^ [64]. Here, CCCP treatment was found to promote increased NF-κB activation through the control of ROS production while suppressing the expression of HO-1, suggesting that antioxidant activity is impaired while inflammatory and pro-oxidant responses are enhanced. Control MC3T3-E1 grown on titanium plates exhibited low levels of NF-κB expression, while these levels rose significantly after CCCP treatment with concomitant increases in Bax expression and Bcl-2 downregulation consistent with the relationship between NF-κB and both inflammation and OS.

Animal studies were performed to further examine the impact of NaB on titanium nail osseointegration, using OVX rats to simulate the estrogen deficiency present in PMO patients. These rats were then implanted with titanium nails in an effort to mimic a post-fracture surgical state, and they were subsequently orally treated using NaB. Micro-CT scans of 0.25 mm of the bone surrounding implanted titanium nails were used to evaluate bone formation around the nail tip, while H&E staining was used to assess bone trabeculae number, spacing, and thickness. Masson’s staining was further employed to quantify new bone formation around these nails, and a pullout force assay was performed to test the resistance of these nails to extraction. Relative to normal control rates, those in the PMO model group exhibited reduced bone mass and fewer bone trabeculae and new bone surrounding implanted titanium nails, with nails being removed relatively easily in pullout force testing. NaB treatment partially reversed these deleterious changes, although they were not restored to the levels observed in healthy control rats. While these results are promising, they are subject to some limitations. For one, osteoclast protein expression was not examined in sufficient detail, and NOX4 may be expressed at higher levels in osteoclasts than in osteoblasts. Such NOX4 expression may represent a key mechanism through which osteoclasts can promote OS. It was also not possible to place cells on titanium plates for histological or immunofluorescent staining assays, precluding efforts to fully simulate in vivo conditions. Future studies will be performed with the goal of correcting these deficiencies.

## Conclusion

In conclusion, these results demonstrate that estrogen deficiency can contribute to elevated ROS levels, in turn suppressing the normal activity and function of osteoblasts and compromising normal bone formation, decreasing the bone mass surrounding implanted titanium nails in PMO patients undergoing surgical fracture treatment. The administration of NaB after surgery in these patients can reverse these effects by decreasing ROS levels, thereby enhancing osteoblast function to facilitate better bone formation and osseointegration. However, owing to the relatively short study period, the low number of tested animals, and certain limitations to the utilized cell models, additional research will be vital to further refine and expand on these analyses.

## Data Availability

The datasets used and analyzed during the current study are available from the corresponding author upon reasonable request.

## Abbreviations

NaB: Sodium butyrate
ROS: Reactive oxygen species
PMA/TPA: Phorbol 12-myristate 13-acetate
OVX: Bilateral ovariectomized
CCCP: Carbonyl cyanide 3-chlorophenylhydrazone
Micro-CT: Micro-computed tomography
HE: Hematoxylin–eosin
MDA: Malondialdehyde
SOD: Superoxide dismutase
Nrf2: Nuclear factor erythroid 2-related factor 2
ALP: Alkaline phosphatase
OCN: Osteocalcin
COLL 1: Type 1 collagen
BMP2: Bone morphogenetic protein-2
PKC: Protein kinase C
PKCα: Protein kinase C alpha
NOX4: NADPH oxidase 4 proteins
NF-κB: Nuclear factor-kappa B
HO-1: Heme oxygenase 1

## References

[1] Clynes MA, Harvey NC, Curtis EM (2020). The epidemiology of osteoporosis. Br Med Bull.

[2] Curtis EM, Moon RJ, Harvey NC (2017). The impact of fragility fracture and approaches to osteoporosis risk assessment worldwide. Bone.

[3] Sugimoto T, Sato M, Dehle FC (2016). Lifestyle-related metabolic disorders, osteoporosis, and fracture risk in Asia: a systematic review. Value Health Reg Issues.

[4] Hernlund E, Svedbom A, Ivergård M (2013). Osteoporosis in the European Union: medical management, epidemiology and economic burden. A report prepared in collaboration with the international osteoporosis foundation (IOF) and the European federation of pharmaceutical industry associations (EFPIA). Arch Osteoporos.

[5] Apostu D, Lucaciu O, Berce C (2018). Current methods of preventing aseptic loosening and improving osseointegration of titanium implants in cementless total hip arthroplasty: a review. J Int Med Res.

[6] Marco F, Milena F, Gianluca G (2005). Peri-implant osteogenesis in health and osteoporosis. Micron Oxford, England.

[7] Du Z, Chen J, Yan F (2009). Effects of Simvastatin on bone healing around titanium implants in osteoporotic rats. Clin Oral Implant Res.

[8] Wu JC, Huang WC, Tsai HW (2011). Pedicle screw loosening in dynamic stabilization: incidence, risk, and outcome in 126 patients. Neurosurg Focus.

[9] Aro HT, Alm JJ, Moritz N (2012). Low BMD affects initial stability and delays stem osseointegration in cementless total hip arthroplasty in women: a 2-year RSA study of 39 patients. Acta Orthop.

[10] Boller S, Jahnke A, Augustin L (2019). Age-related osseointegration of a short hip stem: a clinical and radiological 24 months follow-up. Arch Orthop Trauma Surg.

[11] Brecht JG, Kruse HP, Möhrke W (2004). Health-economic comparison of three recommended drugs for the treatment of osteoporosis. Int J Clin Pharmacol Res.

[12] Blake J, Cosman FA, Lewiecki EM, McClung MR, Pinkerton J, Shapiro M (2021). Management of osteoporosis in postmenopausal women: the 2021 position statement of The North American Menopause society. Menop J North Am Menop Soc.

[13] Rosini S, Rosini S, Bertoldi I (2015). Understanding bisphosphonates and osteonecrosis of the jaw: uses and risks. Eur Rev Med Pharmacol Sci.

[14] Shannon J, Shannon J, Modelevsky S (2011). Bisphosphonates and osteonecrosis of the jaw. J Am Geriatr Soc.

[15] Shibahara T (2019). Antiresorptive agent-related osteonecrosis of the jaw (ARONJ): a twist of fate in the bone. Tohoku J Exp Med.

[16] Li DY, Yu JC, Xiao L (2017). Autophagy attenuates the oxidative stress-induced apoptosis of Mc3T3-E1 osteoblasts. Eur Rev Med Pharmacol Sci.

[17] Kimball JS, Johnson JP, Carlson DA (2021). Oxidative stress and osteoporosis. J Bone Joint Surg Am.

[18] Zhao F, Guo L, Wang X (2021). Correlation of oxidative stress-related biomarkers with postmenopausal osteoporosis: a systematic review and meta-analysis. Arch Osteoporos.

[19] Fan ZQ, Bai SC, Xu Q (2021). Oxidative stress induced osteocyte apoptosis in steroid-induced femoral head necrosis. Orthop Surg.

[20] Agidigbi TS, Kim C (2019). Reactive oxygen species in osteoclast differentiation and possible pharmaceutical targets of ROS-mediated osteoclast diseases. Int J Mol Sci.

[21] Rai D, Tripathi AK, Sardar A (2022). A novel BMP2 secretagogue ameliorates glucocorticoid induced oxidative stress in osteoblasts by activating NRF2 dependent survival while promoting Wnt/β-catenin mediated osteogenesis. Free Radical Biol Med.

[22] Liu HD, Ren MX, Li Y (2022). Melatonin alleviates hydrogen peroxide induced oxidative damage in MC3T3-E1 cells and promotes osteogenesis by activating SIRT1. Free Radic Res.

[23] Qin D, Zhang H, Zhang H (2019). Anti-osteoporosis effects of osteoking via reducing reactive oxygen species. J Ethnopharmacol.

[24] Cheng J, Wang H, Zhang Z (2019). Stilbene glycoside protects osteoblasts against oxidative damage via Nrf2/HO-1 and NF-κB signaling pathways. Arch Med Sci.

[25] Saito N, Mikami R, Mizutani K (2022). Impaired dental implant osseointegration in rat with streptozotocin-induced diabetes. J Periodontal Res.

[26] Tu Y, Yang R, Xu X (2021). The microbiota-gut-bone axis and bone health. J Leukoc Biol.

[27] Zhang YW, Li YJ, Lu PP (2021). The modulatory effect and implication of gut microbiota on osteoporosis: from the perspective of "brain-gut-bone" axis. Food Funct.

[28] Hansen MS, Frost M (2022). Alliances of the gut and bone axis. Semin Cell Dev Biol.

[29] Ferrario C, Taverniti V, Milani C (2014). Modulation of fecal Clostridiales bacteria and butyrate by probiotic intervention with Lactobacillus paracasei DG varies among healthy adults. J Nutr.

[30] Dou X, Gao N, Yan D, Shan A (2020). Sodium butyrate alleviates mouse colitis by regulating gut microbiota dysbiosis. Animals.

[31] Wang W, Fang D, Zhang H (2020). Sodium butyrate selectively kills cancer cells and inhibits migration in colorectal cancer by targeting thioredoxin-1. Onco Targets Ther.

[32] Iwami K, Moriyama T (1993). Effects of short chain fatty acid, sodium butyrate, on osteoblastic cells and osteoclastic cells. Int J Biochem.

[33] Katono T, Kawato T, Tanabe N (2008). Sodium butyrate stimulates mineralized nodule formation and osteoprotegerin expression by human osteoblasts. Arch Oral Biol.

[34] Han J, Yang K, An J (2022). The role of NRF2 in bone metabolism—friend or foe?. Front Endocrinol (Lausanne).

[35] Zhang K, Meng M, Gao L (2018). Sodium butyrate improves high-concentrate-diet-induced impairment of ruminal epithelium barrier function in goats. J Agric Food Chem.

[36] Volpe CMO, Villar-Delfino PH, Dos Anjos PMF (2018). Cellular death, reactive oxygen species (ROS) and diabetic complications. Cell Death Dis.

[37] Kang Q, Yang C (2020). Oxidative stress and diabetic retinopathy: molecular mechanisms, pathogenetic role and therapeutic implications. Redox Biol.

[38] Bär L, Hase P, Föller M (2019). PKC regulates the production of fibroblast growth factor 23 (FGF23). PLoS ONE.

[39] Huang W, Shang WL, Li DH (2012). Simvastatin protects osteoblast against H2O2-induced oxidative damage via inhibiting the upregulation of Nox4. Mol Cell Biochem.

[40] Lepetsos P, Papavassiliou KA, Papavassiliou AG (2019). Redox and NF-κB signaling in osteoarthritis. Free Radical Biol Med.

[41] Diaz-Meco MT, Moscat J (2012). The atypical PKCs in inflammation: NF-κB and beyond. Immunol Rev.

[42] Toriumi K, Horikoshi Y, Osamura RY, Yamamoto Y, Nakamura N, Takekoshi S (2013). Carbon tetrachloride-induced hepatic injury through formation of oxidized diacylglycerol and activation of the PKC/NF-κB pathway. Lab Invest.

[43] Chen G, Ran X, Li B (2018). Sodium butyrate inhibits inflammation and maintains epithelium barrier integrity in a TNBS-induced Inflammatory bowel disease mice model. EBioMedicine.

[44] Okubo R, Sanada LS, Castania VA (2017). Jumping exercise preserves bone mineral density and mechanical properties in osteopenic ovariectomized rats even following established osteopenia. Osteoporos Int.

[45] Melton LJ, Thamer M, Ray NF (1997). Fractures attributable to osteoporosis: report from the national osteoporosis foundation. J Bone Miner Res.

[46] Wang L, Yu W, Yin X (2021). Prevalence of osteoporosis and fracture in China: the China osteoporosis prevalence study. JAMA Netw Open.

[47] Mücke T, Krestan CR, Mitchell DA (2016). Bisphosphonate and medication-related osteonecrosis of the jaw: a review. Semin Musculoskelet Radiol.

[48] Jones CB (2012). Augmentation of implant fixation in osteoporotic bone. Curr Osteoporos Rep.

[49] Tang X, Ma S, Li Y (2020). Evaluating the activity of sodium butyrate to prevent osteoporosis in rats by promoting osteal GSK-3β/Nrf2 signaling and mitochondrial function. J Agric Food Chem.

[50] Takuwa Y, Ohse C, Wang EA (1991). Bone morphogenetic protein-2 stimulates alkaline phosphatase activity and collagen synthesis in cultured osteoblastic cells, MC3T3-E1. Biochem Biophys Res Commun.

[51] An J, Yang H, Zhang Q (2016). Natural products for treatment of osteoporosis: the effects and mechanisms on promoting osteoblast-mediated bone formation. Life Sci.

[52] Rogers MB, Shah TA, Shaikh NN (2015). Turning bone morphogenetic protein 2 (BMP2) on and off in mesenchymal cells. J Cell Biochem.

[53] Raje MM, Ashma R (2019). Epigenetic regulation of BMP2 gene in osteoporosis: a DNA methylation study. Mol Biol Rep.

[54] Shi S, Kirk M, Kahn AJ (1996). The role of type I collagen in the regulation of the osteoblast phenotype. J Bone Miner Res.

[55] Lu QB, Du Q, Wang HP (2020). Salusin-β mediates tubular cell apoptosis in acute kidney injury: Involvement of the PKC/ROS signaling pathway. Redox Biol.

[56] Qin J, Peng Z, Yuan Q (2019). AKF-PD alleviates diabetic nephropathy via blocking the RAGE/AGEs/NOX and PKC/NOX Pathways. Sci Rep.

[57] Caspa Gokulan R, Devaraj H (2021). Stem cell markers CXCR-4 and CD133 predict aggressive phenotype and their double positivity indicates poor prognosis of oral squamous cell carcinoma. Cancers.

[58] Zhou Z, Xu N, Matei N (2021). Sodium butyrate attenuated neuronal apoptosis via GPR41/Gβγ/PI3K/Akt pathway after MCAO in rats. J Cereb Blood Flow Metabol.

[59] Li D, Bai X, Jiang Y (2021). Butyrate alleviates PTZ-induced mitochondrial dysfunction, oxidative stress and neuron apoptosis in mice via Keap1/Nrf2/HO-1 pathway. Brain Res Bull.

[60] Chen JR, Lazarenko OP, Blackburn ML (2022). Nox4 expression in osteo-progenitors controls bone development in mice during early life. Commun Biol.

[61] Das R, Xu S, Quan X (2014). Upregulation of mitochondrial Nox4 mediates TGF-β-induced apoptosis in cultured mouse podocytes. Am J Physiol Renal Physiol.

[62] Morgan MJ, Liu ZG (2011). Crosstalk of reactive oxygen species and NF-κB signaling. Cell Res.

[63] Keum YS, Choi BY (2014). Molecular and chemical regulation of the Keap1-Nrf2 signaling pathway. Molecules.

[64] Saha S, Buttari B, Panieri E, Profumo E, Saso L (2020). An overview of Nrf2 signaling pathway and its role in inflammation. Molecules.

